# Population Pharmacokinetics of Clozapine and Norclozapine and Switchability Assessment between Brands in Uruguayan Patients with Schizophrenia

**DOI:** 10.1155/2019/3163502

**Published:** 2019-03-06

**Authors:** Ismael Olmos, Manuel Ibarra, Marta Vázquez, Cecilia Maldonado, Pietro Fagiolino, Gustavo Giachetto

**Affiliations:** ^1^Pharmacy Department, Vilardebó Hospital, Avenida Millán 2515, 11800 Montevideo, Uruguay; ^2^Pharmaceutical Sciences Department, Faculty of Chemistry, Universidad de la República, Avenida General Flores 2124, P. O. Box 1157, 11800 Montevideo, Uruguay; ^3^C Pediatrics Clinics, Pereira Rossell Hospital, Bulevar Gral. Artigas 1550, 11600 Montevideo, Faculty of Medicine, Universidad de la República, Uruguay

## Abstract

Clozapine (CZP) is an atypical antipsychotic agent commonly used in the treatment of schizophrenia. It is metabolized primarily by CYP1A2 enzyme, yielding a pharmacologically active metabolite, norclozapine (NCZP). Significant intra- and interindividual pharmacokinetic (PK) variability for CZP and NCZP has been observed in routine therapeutic drug monitoring. So the goal of this study was to evaluate the magnitude and variability of concentration exposure to CZP and its active metabolite NCZP on pharmacokinetic parameters in Uruguayan patients with schizophrenia with a focus on covariates such as cigarette smoking, age, sex, caffeine consumption, brands available of CZP, and comedication using population PK (PPK) modeling methodologies. Patients with a diagnosis of schizophrenia treated with brand-name CZP (Leponex®) for more than a year were included in the study. Then these patients were switched to the similar brand of CZP (Luverina®). Morning predose blood samples for determination of CZP and NCZP using a HPLC system equipped with a UV detector were withdrawn on both occasions at steady state and under the same comedication. Ninety-eight patients, 22 women and 76 men, took part in the study. Mean ± standard deviation for CZP and NCZP concentration was 421 ± 262 ng/mL and 275 ± 180 ng/mL, respectively. After covariate evaluation, only smoking status remained significant in CZP apparent clearance, inducing a mean increment of 32% but with no clinical impact. The results obtained with the two brands of CZP should ensure comparable efficacy and tolerability with the clinical use of either product. Smoking was significantly associated with a lower exposure to CZP due to higher clearance. The results obtained with the two brands commercialized in our country hint a bioequivalence scenario in the clinical setting.

## 1. Introduction

Schizophrenia is a severe neuropsychiatric disorder characterized by a high degree of morbidity and mortality with a prevalence rate from 0.4 to 1.4 % of the population [1]. Clozapine (CZP), a tricyclic dibenzodiazepine, is a second-generation antipsychotic drug primarily used in the treatment of schizophrenia and bipolar disorder [2–4]. CZP is classified as an “atypical” antipsychotic drug because its profile of binding to dopamine receptors and its effects differ from those exhibited by more typical antipsychotic drug products. In particular, CZP has low affinity for D1, D2, D3, and D5 receptors and has a high affinity for the D4 receptor. This evidence may explain the relative freedom of CZP from extrapyramidal side effects. CZP also acts as an antagonist at adrenergic, cholinergic, histaminergic, and serotonergic receptors [5].

From the pharmacokinetic (PK) point of view, the absorption of CZP is almost complete, with an oral bioavailability between 60 and 70% due to first pass metabolism. Food does not seem to affect the amount of drug absorbed. The time to peak concentration after oral dosing is about 2.5 hours. The elimination half-life is about 14 hours at steady-state conditions, plasma clearance between 8.7 and 53.3 L/h, and distribution volume between 1.6 and 7.3 L/kg. CZP is 95 % bound to plasma proteins, primarily alpha-1-glycoprotein [5–7].

CZP undergoes extensive metabolism principally to the stable metabolites, desmethylclozapine (active metabolite) and clozapine N-oxide. CYP1A2 and to a lesser extent CYP3A4 catalyze the demethylation of CZP while N-oxide formation is catalyzed by CYP3A4. The isoenzymes CYP2D6, 2C9, and 2C19 appear to play minor roles as well [8–10]. In man, CZP can also be metabolized to a reactive intermediate by hepatic P450 enzymes, myeloid cells, and peripheral blood polymorphonuclear leukocytes [11, 12]. Myeloperoxidase, the major enzyme present in polymorphonuclear leukocytes can bioactivate CZP to a radical cation and then to a nitrenium ion. The latter has been implicated in the pathogenesis of agranulocytosis [13]. Approximately 0.8% of patients treated with CZP develop agranulocytosis and puts individuals at risk of severe infections, which are often fatal. However, mandatory monitoring of hematological parameters has decreased the incidence of agranulocytosis and increased patient safety [14, 15].

Both CZP and norclozapine (NCZP, its major metabolite) predict the clinical outcome [16]. Thus, routine therapeutic drug monitoring (TDM) of CZP and NCZP is recommended to ensure safety and avoid toxic adverse events [17]. Although the effective CZP plasma levels remain debated, most researchers find that a therapeutic window of 350-600 ng/mL for CZP plasma concentrations is associated with an increased probability of a good clinical response to the drug [18]. Concentrations higher than 1000 ng/mL could increase risk of seizures. NCZP levels are generally in a range from 50 to 90% of the concentrations of CZP. The lower limit of CZP may be 200 ng/mL once control is achieved or in elderly patients [17].

There is evidence of P-glycoprotein involvement in CZP absorption across the intestine and the blood brain barrier (BBB), but according to some authors olanzapine and risperidone are the only antipsychotic agents that may inhibit P-gp activity in the BBB [19, 20].

Despite the effectiveness of CZP as a standard drug for schizophrenia treatment, significant intra- and interindividual pharmacokinetic variability for CZP and even for NCZP has been observed in routine TDM [21, 22].

The influence of sex, smoking, and other factors on CZP and/or NCZP plasma concentrations has been previously reported, though little information is available about the influence of these factors on the Hispanic population [23–26].

In Uruguay, two brands of CZP were registered by the Health Ministry and are now available for use in our population: the brand-name drug (Leponex®, Novartis Laboratories) and the similar formulation (Luverina®, Celsius Laboratories), both of them containing 100 mg of CZP. Bioequivalence studies are required for drugs having a narrow therapeutic index; however, only a few studies have been carried out since the Uruguayan regulation was approved in 2007. CZP is among these drugs and its formulations have not been studied in vivo yet to demonstrate bioequivalence. For this reason, concern has arisen among psychiatrists when switching from one brand to the other, as this could have significant clinical implications. Initial in vitro evaluation of similar and brand-name drugs in USP-2 Apparatus (paddles) at WHO's biorelevant [27] media (pH 1.2, 4.5 and 6.8), simulating gastrointestinal physiological in vivo conditions, can anticipate the behavior of problematic products.

The main objective of this study was to evaluate, using population PK modeling methodologies, the magnitude and variability of concentration exposure to CZP and its active metabolite NCZP on PK parameters in Uruguayan patients with schizophrenia with a focus on cigarette smoking, age, sex, caffeine consumption, and comedication. In addition, the switchability of CZP brands available in Uruguay in the clinical setting was assessed.

## 2. Materials and Methods

### 2.1. Patients and Data Collection

The inclusion criteria for patients included a diagnosis of schizophrenia according to the Diagnostic and Statistical Manual of Mental Disorders IV (DSM-IV) carried out by the attending physicians of the psychiatric hospital in Uruguay (Hospital Vilardebó). All patients were treated with the same brand of CZP (Leponex®) for more than a year. Then these patients were switched to Luverina® as the hospital purchase changed CZP brand. Patients were for two months on Luverina® before blood sampling. So morning predose blood samples for determination of CZP and NCZP were withdrawn on both occasions at steady state and under the same comedication. Oral CZP was administered twice a day with each brand. The study conformed to standards indicated by the Declaration of Helsinki and its later amendments, approval was provided by the Ethics Committee of Hospital Vilardebó, and all patients in the study gave written informed consent prior to participation.

Demographic data including sex, age, bodyweight, medication history, dosage regimen, time of last dose, sampling time, concentrations of CZP and NCZP for both brands, smoking habit, information on concomitant medications, caffeine consumption as well as biochemical and hematological test results, and other relevant data were collected using a data collection form.

### 2.2. CZP and NCZP Determination

Predose morning blood samples were withdrawn and placed in heparinized tubes. Plasma was separated by centrifugation and stored at -25°C until analysis. Drug quantification in plasma was performed using a validated method with minor modifications [28]. A Shimadzu LC-6A high-performance liquid chromatography system equipped with a UV Shimadzu SPD-6A detector was used. Fifty microliters of internal standard (Medazepam 16 *μ*g/mL in methanol) were added to 1.0 mL of plasma. Then 500 *μ*L of sodium carbonate 1 M and some drops of t-butanol were added and the extraction was performed with 6 mL of cyclohexane and vortex shaken for 1 minute. After centrifugation the supernatant was separated and dried under nitrogen stream at 37-40°C. Dry residue was dissolved with 50 *μ*L of mobile phase and 20 *μ*L injected into the equipment. The separation of the compounds was performed on a Phenomenex Luna C18 column (5 *μ*m, 100 A, 150 mm × 4.6 mm, id). The mobile phase consisted of methanol:acetonitrile: buffer phosphate 50 mM pH 6.5 (20:20:60) and was pumped at a flow-rate of 1.6 mL/min. The column compartment was kept at 36°C and detection was performed at the wavelength of 230 nm. Under these conditions the retention times of analytes were 6.6, 8.2, and 9.5 min for NCZP, CZP, and medazepam, respectively. The height ratios of the compounds' peaks to the medazepam peak (internal standard) were employed for all calculations. The HPLC method was linear between 54.8 (lower limit of quantification: LLOQ) and 1086 ng/mL for CZP and 72.3 (LLOQ) and 1085 ng/mL for NCZP. Inter and intraday precision and accuracy were below 15% for both compounds.

### 2.3. Population Pharmacokinetic Modeling

The PPK analysis was performed using NONMEM® 7.4 (ICON plc.) together with the modeling and simulation workbench Pirana-PsN-Xpose [29]. Given the very sparse nature of the data, several assumptions were made with the purpose of estimate population means and between-subject variability for CZP and NCZP apparent clearance (CL/F). A simultaneous approach was implemented for evaluation of parent and metabolite observations. The structural model was defined with a one-compartment disposition for both substances, fixing the apparent volumes of distribution (V/F) of 750 L and 1860 L for CZP and NCZP, respectively. These mean values were estimated by Golden and Honigfeld after conducting a multiple dosing bioequivalence study with extensive sampling in 30 patients with schizophrenia and were attributed in this work to a 70-kg body weight subject under a proportional centered model:

(1) $$\begin{array}{c} V_{i}=V*\frac{{BW}_{i}}{70} \end{array}$$

where Vi is the apparent volume of distribution for the i-th subject, BWi its body weight in kilograms, and V the apparent volume of distribution for a 70-kg body weight subject [30].

CZP first-order constant rate for absorption (ka) was fixed to a value of 1.24 h^−1^ as estimated by Jerling et al. [31]. Both CZP and NCZP elimination were regarded as first-order kinetic processes. Complete conversion of CZP into NCZP was assumed and a factor was included in NCZP formation to account for the molecular weight differences. These assumptions allow identifiability of apparent clearance of clozapine (CLap_CZP_ = CL_CZP_/F) and norclozapine (CLap_NCZP_ = CL_NCZP_/F/f), where F is the oral bioavailability of CZP and f the fraction of NCZP formed after CZP biotransformation (fixed to 1). F was fixed to 1 for Leponex® and evaluated for Luverina® as an estimate of the relative CZP bioavailability between both drug products. To account for the effect of body weight on clearance, a power model was included evaluating different coefficients and finally fixing this value to the allometric standard of 0.75; therefore,

(2) $$\begin{array}{c} {CLap}_{i}=CLap*{\left(\;\frac{{BW}_{i}}{70}\;\right)}^{0.75} \end{array}$$

The pharmacokinetic parameters were assumed to follow a log-normal distribution, including the between-subject variability with an exponential model:

(3) $$\begin{array}{c} \theta_{i}=\theta_{pop}*e^{\eta_{i}} \end{array}$$

where *θ*_*pop*_ stands for the mean population estimate, *θ*_*i*_ the estimate for the i-th subject, and *η*_*i*_ the difference between *θ*_*i*_ and *θ*_*pop*_ in the log-scale. Log-normal distribution is given by assuming *η* ~ *N*(0, *ω*^2^), where *ω*^2^ is the between-subject variability.

Interoccasion variability was not identifiable since only one observation was available per subject at each period (under treatment with Leponex® or Luverina®) and thus was not included. Residual unexplained variability was modeled with a proportional error:

(4) $$\begin{array}{c} C_{ik}=C_{pred}*\left(\;1+\varepsilon_{ik}\;\right) \end{array}$$

*C*_*ik*_ is the predicted trough concentration for the i-th subject under treatment k, *C*_*pred*_ is the population prediction, and *ε*_*ik*_ is the proportional residual variability assumed to be normally distributed around zero with variance *σ*^2^.

Covariate search was performed for CLap_CZP_, CLap_NCZP_, and F, evaluating the effect of sex, smoking status, CZP formulation, beginning of treatment, caffeine consumption, and concomitant treatments: valproic acid, benzodiazepines, antidepressants, antipsychotics, antidiabetics, and oral hypoglycemic drugs. The effect of daily CZP dose on both apparent clearances was also assessed. Covariate effect was evaluated separately for each possible source of variability, including as a second step in a full-model those factors which significantly reduced the objective function (OFV) considering a chi-square distribution for the OFV difference (Δ_*OFV*_ = 3.84, p<0.05 for 1 df) and finally performing a backward elimination with a stricter p value of 0.001 (Δ_*OFV*_ = 10.83 for 1 df), conserving only those factors with significant contribution to the overall variability. Graphic diagnostics were also obtained for the assessment of covariate inclusion and correlation between covariates. Numerical predictive check (NPC) and Numerical Predictive Distribution Errors (NPDE) versus predictions were used for assessing the final model. The NPC evaluates model misspecification at several percentiles of data distribution by contrasting the observed data with nonparametric confidence intervals obtained with data simulated by the model. The percentages of observed data above the upper and below the lower limit of a prediction interval built from these simulations are divided by the corresponding expected percentage (i.e., for a 90% prediction interval, 5% of the data is expected to be found above the upper limit). The difference between these ratios and the unity (ideal value) is statistically evaluated throughout a coverage plot, allowing the assessment of the model predictions. NPDE is also a simulation-based diagnostic which estimates model prediction discrepancies with the observed data. A model describes the data well when the prediction discrepancies are evenly distributed [32]. For estimation of parameter precision, the final model was bootstrapped with 200 samples and nonparametric confidence intervals were obtained from the bootstrapped distribution.

### 2.4. In Vitro Dissolution Study

The in vitro dissolution was performed at the following 3 media: (1) HCl/KCl pH 1.2 solution, (2) acetate buffer pH 4.5, and (3) phosphate buffer pH 6.8. Six units of each product were tested in Distek® dissolution system 2100C equipment. The conditions were USP-2 Apparatus according to WHO guidelines for biowaivers, 75 rpm stirring speed; 900mL of medium per vessel maintained at 37 ± 0.5°C. Samples were automatically withdrawn by the use of an Agilent 89092EO pump at: 5, 10, 15, 30, 45, 60 minutes. The drug release at different time intervals was measured by UV-visible spectrophotometer at 230 nm (Agilent 8453 and ChemStation® software). Cumulative percentages of dose dissolved were calculated.

## 3. Results 

The final dataset included 98 Caucasian patients, 22 women and 76 men. A total of 171 trough observations were recorded for both CZP and NCZP, of which 146 corresponded to 73 patients who completed both periods receiving Luverina® and Leponex®. Sixty-eight (93%) of these patients conserved the dosage regime after the change of brand. For 25 patients, only one period was available: 17 under treatment with Luverina® and 8 under treatment with Leponex®. Demographic data and other characteristics of the patients for both brands as well as CZP and NCZP concentrations are summarized in Table 1. Experimental concentrations for CZP were all above the LLOQ, while NCZP left-censored data represented less than 4% of total observations and was therefore included as such in the population analysis. Daily dose of CZP varied between 150 and 700 mg. There was large between-subject variability in CZP and NCZP plasma concentrations. Mean ± standard deviation for CZP and NCZP concentration was 421 ± 262 ng/mL and 275 ± 180 ng/mL, respectively.

**Figure 1:**
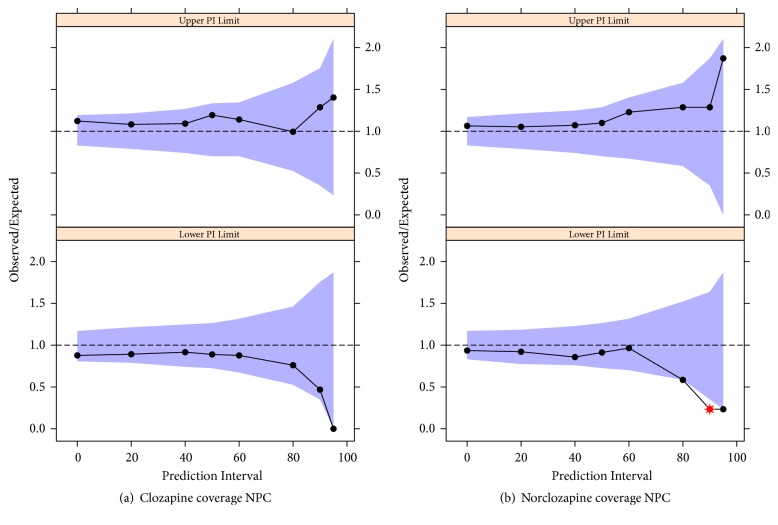
Numerical predictive check (NPC) coverage plots for CZP and NCZP plasma concentrations. The plot shows the ratios between observed and expected percentages of data above the upper and below the lower limits of the 0%, 20%, 40%, 50%, 60%, 80%, 90%, and 95% prediction intervals (black dots), and their corresponding predicted distribution as 95% confidence intervals (blue area). Outliers are shown as red dots.

**Figure 2:**
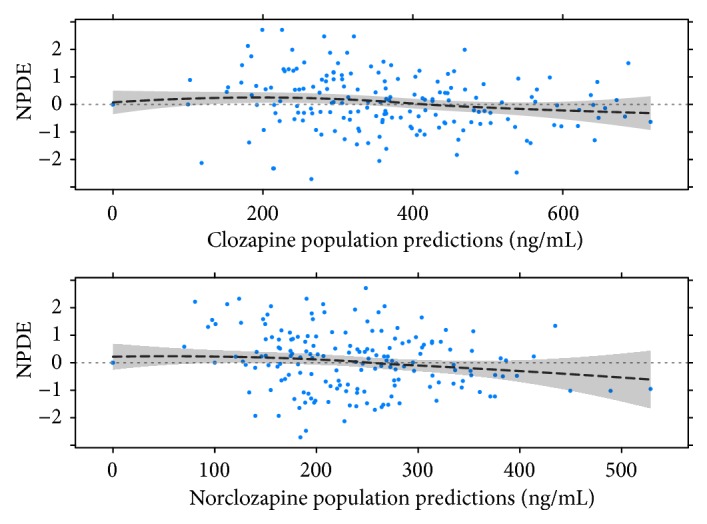
Normalized prediction distribution errors (NPDE) versus CZP (above) and NCZP (below) population predictions.

The final model estimates are shown in Table 2, while diagnostic graphs NPC and NPDE are included in Figures 1 and 2, respectively. All parameters were estimated with an acceptable uncertainty. NPDE versus predicted CZP and NCZP concentrations (Figure 2) show data points mainly contained between -1.96 and 1.96 and evenly distributed around the horizontal zero-line, as expected for a good fit. In the NPC coverage plot (Figure 1) no major trends are observed and almost all ratios did not significantly differ from the unity. The outlier observed for NCZP at the lower limit of the prediction intervals was not regarded as indicator of model misspecification. The results for CZP and NCZP mean apparent clearances are in accordance with previous studies performed in different populations of patients with schizophrenia [30, 31, 33]. In the same way, high between-subject variability was observed for all estimates pharmacokinetic parameters. No correlation was observed between CZP and NCZP apparent clearances with the daily dose.

After covariate evaluation, only smoking status remained significant in CZP clearance (p<0.05), inducing a mean increment of 32%. Sex did not produce a significant impact in these data: an increase in the AIC was observed after including it as a covariate on CZP and NCZP apparent clearance, and no significant differences between male and female estimates were obtained. This covariate was reevaluated after smoking factor was included to discard a masking effect, obtaining similar results. Smoking subjects represented 45% within each sex. The inclusion of caffeine intake as a covariate on compound elimination was also discarded, after estimating a very small (less than 1% increase) impact on CZP and NCZP CLap. Regarding drug-drug interactions, the most influential coadministered drug was valproic acid (VPA), increasing NCZP CLap by 10%, a relationship that was not retained for the final model because it was not found to be statistically significant. Among all subjects in the study, 28 also received antidepressants, sertraline, or escitalopram. However, no impact on CZP and/or NCZP CLap was observed with this medication. Inclusion of between-subject variability for the bioavailability factor significantly improved the fit. The relative bioavailability of Luverina® versus Leponex® was estimated as 0.892. The bootstrapped 95% confidence interval for the relative bioavailability, an estimate of bioequivalence ratio of means, was 0.769-1.02. The same adverse events (weight gain and sialorrhea) were reported for both brands of CZP.

**Figure 3:**
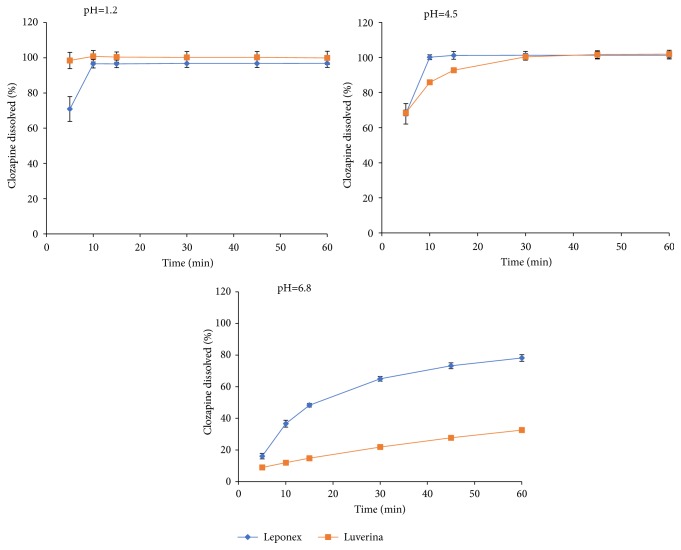
In vitro dissolution profiles (mean ± standard deviation) of clozapine at pH 1.2, 4.5, and 6.8.

Mean dissolution profiles of both formulations at the three different media (pH 1.2, 4.5, and 6.8) are shown in Figure 3.

## 4. Discussion

Mean plasma concentrations of CZP and NCZP were within the therapeutic concentrations reported in the literature and mentioned in Introduction section.

Smoking incidence on CZP clearance has been reported before with a similar magnitude of effect for different populations of patients [22, 23, 33].

Analysis of the plasma CZP: NCZP ratio can give us information about the metabolic status of CZP and patient's adherence [17]. In our study CZP: NCZP ratio ± standard deviation for smokers and no smokers was 1.64 ± 1.25 and 2.30 ± 1.20, respectively. The significant difference between both ratios (p<0.01) is in accordance with the significant different CZP clearance found in this work. There was no significant difference between the median daily oral CZP dose for smokers and no smokers: 350 mg (range 150-700 mg) and 400 mg (200-650 mg), respectively. Smoking status was previously identified as a statistically significant covariate affecting the apparent clearance of CZP and NCZP [34]. Because the activity of CYP1A2 is greater in smokers than in nonsmokers and this enzyme is involved in CZP and NCZP metabolism, CLap_CZP_ and CLap_NCZP_ are likely influenced by smoking status [35]. Nevertheless in our study only CLap_CZP_ seemed to be affected. Interpreting our data correctly if CLap_NCZP_ remained unchanged, an increase in both NCZP bioavailability and clearance would be the reason for this observation. Surprisingly, smokers were not exposed to larger dosages due to the higher apparent clearance of CZP. It is likely that the induction found in this work did not have a clinical impact. One limitation of the study is that smoking status was assessed using patient self-reporting. We dichotomized patients into smokers and nonsmokers but did not assess the magnitude of smoking. Some authors found that a daily consumption of 7-12 cigarettes is probably sufficient for maximum induction of CZP metabolism in 50 % [36]. The increase in CLap_CZP_ observed in this work for smokers was 32 %. This increase could be because of an increase in systemic elimination or a decrease in bioavailability secondary to an induction in presystemic metabolism. As the isoenzyme CYP1A2 is practically not detected in the intestine, only an increase in systemic elimination could explain our results [37].

Although sex was also reported to be a significant covariate affecting the apparent clearance of CZP and NCZP explained by a different CYP1A2 activity between men and women, our study found no differences in the CLap_CZP_ between male and female [22–25]. According to some authors not even the CYP1A2 genetic polymorphism seemed to have significant clinical effect on sex differences and their results also showed that CZP clearance is strongly associated with smoking behavior [38]. Moreover, although there is a sex imbalance in the number of participants (more men), the small difference observed between point estimates for Clap when sex was assessed as a covariate enables us to affirm that there are no clinical sex-related differences in this population.

Antidepressants are frequently used in the treatment of depressive symptoms associated with schizophrenia. In patients taking CZP, choice of antidepressant is complicated by additive pharmacodynamics effects and by pharmacokinetic interactions. The various antidepressants differ in their potency to inhibit CYP enzymes [39, 40]. Previous reports have shown that fluvoxamine can increase plasma CZP concentrations by inhibition of cytochrome P450 (CYP) 1A2 [41]. Citalopram, escitalopram, and sertraline do not elevate plasma CZP levels when these drugs are coadministered because they do not inhibit the relevant enzyme systems involved in CZP metabolism (CYP3A4 and CYP1A2). As it was stated before, the patients in our study that were under antidepressant therapy were taking sertraline or escitalopram, so no overall change in mean CZP levels due to these medications should be expected.

Coadministration of VPA is very common in patients under CZP treatment as CZP can trigger convulsive seizures. 40 % of the patients in this study were treated with VPA at low doses (approximately 400 mg/day). No influence of VPA on the metabolism of CZP was found in this study. Literature had provided contradictory results concerning the effects of VPA on CZP metabolism. Studies with different designs indicated no effects, mild inhibition, or mild induction [42–46]. One finding of the Italian clozapine TDM study was that VPA appeared to potentiate smoking inductive effects on CZP metabolism since smoking alone produced a 20% reduction in plasma CZP concentrations whereas smoking and VPA together produced a 41% reduction [47]. A more plausible explanation for the findings of the Italian group is that the reduction in total CZP concentrations reported could be explained by VPA displacing CZP from the plasma proteins, increasing the free fraction of CZP and subsequently a decrease in CZP serum total concentrations can be observed [48]. However, free drug concentrations of CZP would show a linear kinetics after the equilibrium is reached and no clinical relevance of this interaction should be seen. Due to the low doses of VPA used by the patients in our study, protein binding displacement of CZP was probably not happening. The limitation of our study is that no plasma concentrations of VPA were measured.

It is important to know whether drinking coffee, in the amounts consumed by patients, has a clinically significant effect on steady-state serum CZP concentrations, especially because schizophrenia is linked to high caffeine intake. Some authors indicate that caffeine reduces CZP clearance most likely by inhibiting CYP1A2 [49, 50]. Changes in habitual caffeine intake can therefore explain some of the large kinetic variability found for CZP and may have clinical consequences in certain individuals [49]. More than 76 % of the enrolled patients in our study consumed coffee, showing no significant differences in either CLap_CZP_ or CLap_NCZP_ with nonconsumers in the covariate analysis. Caffeine consumers were similarly distributed among smoking and nonsmoking groups. Clearance estimates for the nonsmoking group (i.e., basal CLap_CZP_ and CLap_NCZP_) were similar to the previously reported, including a controlled multiple-dose bioequivalence study, indicating that the presence of an inhibiting effect of caffeine over CYP1A2 is unlikely [30].

As stated in Introduction section, bioequivalence requirements were not fulfilled for CZP in Uruguay. As a result, the two formulations available for the treatment of schizophrenia and bipolar disorder have never been studied in vivo and psychiatrists are forced to establish drug treatments without knowing drug product interchangeability. For this reason, switching from brand-name (Leponex®) to similar formulation (Luverina®) has raised concerns among physicians and patients as the evidence related specifically to the safety of switching from brand-name to similar CZP is scarce. Only three studies in the literature [51–53] have compared the bioavailability of generic and brand-name CZP in schizophrenic patients. Taking into account this scenario, it is interesting for us to know if there are significant differences between the two brands that are available in our market. The population analysis carried out in this study allowed, based on scarce data, to estimate the mean bioequivalence ratio T / R for the amount absorbed, indicating a probable bioequivalence in the clinical setting between both formulations for this parameter. Due to the limitations of the sampling plan, the absorption rate of CZP obtained for each formulation could not be assessed, so it is not possible to foresee a scenario of complete bioequivalence between these formulations. Nevertheless, no significant difference in predose plasma CZP and NCZP concentration before and after switching in either case was observed. Furthermore, no changes in schizophrenia control and/or adverse events were recorded upon switching from brand to similar CZP. As only one observation (trough) was available for each patient under each treatment, maximum plasma concentration at steady-state (Cmax,ss) and time to maximum plasma concentration (Tmax,ss) could not be estimated and this is a limitation of the study.

Our in vitro dissolution study is in accordance with our in vivo findings. CZP is a weak basic compound with a pKa of 4.5 and it can be classified as a class II drug according to the Biopharmaceutics Classification System, owing to its good permeation properties through biological membranes and low aqueous solubility [54]. Its aqueous solubility is pH-dependent. As it can be observed in Figure 3, the data obtained in the dissolution studies showed similar behavior for both brands of CZP at pH 1.2 and 4.5. However, at a more neutral pH of 6.8, it did show a different behavior. From our results, this difference could not have impacted on the therapeutic response, since it is unlikely to reach that pH at the gastric level, even with the concomitant administration of proton pump inhibitors. In addition, the very rapid dissolutions observed at gastric and duodenal pH would make it unlikely that more transit through the digestive tract would be required for the complete dissolution of the drug in order to be fully available for absorption. Accordingly, it is highly probable that the in vivo dissolution would be similarly completed by the two brands, and from then the same bioavailability for the two formulations would be achieved.

## 5. Conclusions

Covariate evaluation showed that only smoking status remained significant in CZP apparent clearance, inducing a mean increment of 32%. By means of population pharmacokinetics modeling, a comparative study of the bioavailability between these two products in the clinical setting with very sparse data (1 observation per subject per formulation) could be carried out. Given that bioequivalence regulation has confronted several obstacles in our country, this approach could be applied to assess multisource products being currently used, because their unknown characteristics could entail increased therapeutic failures (inefficacy/toxicity). The results obtained with the two brands commercialized in our country hint a bioequivalence scenario in the clinical setting as the same efficacy and the same adverse effects were reported by the clinicians.

## Figures and Tables

**Table 1:** Demographic data and other characteristics of the patients.

	Total	Smoking	Nonsmoking
N	98	46	52

Sex = Male (N)	76	37	39

Age (years)^b^	39 (20-68)	40 (24-67)	37 (20-68)

Weight (kg)^b^	78 (48-137)	78 (48-120)	80 (57-137)

BMI (kg/m^2^)^b^	26 (15-43)	26 (15-42)	27 (21-43)

Dose (mg/day)^b^	350 (150-700)	350 (150-700)	400 (200-650)

Caffeine (N)	75	38	37

Valproic acid (N)	37	14	23

Antidepressants (N)	28	14	14

Benzodiazepines (N)	41	16	25

Mean CZP(ng/mL)^a^	421 (262)	382 (269)	462 (212)

Mean NCZP (ng/mL)^a^	275 (180)	293 (160)	261 (156)

CZP/NCZP^a^	2.00 (1.55)	1.64 (1.25)	2.30 (1.20)

	*Leponex*	*Luverina*	

N	81	90	

Sex = Male (N)	63	68	

Age (years)^b^	38 (20-67)	39 (22-68)	

Weight (kg)^b^	77 (48-136)	82 (54-137)	

BMI ( kg/m^2^)^b^	26 (15-43)	27 (18-43)	

Dose (mg/day)^b^	400 (200-600)	350 (150-700)	

Caffeine (N)	62	62	

Valproic acid (N)	28	32	

Antidepressants (N)	23	28	

Benzodiazepines (N)	35	37	

Mean CZP (ng/mL)^a^	432 (264)	412 (261)	

Mean NCZP (ng/mL)^a^	294 (185)	258 (175)	

CZP/NCZP^a^	2.04 (1.66)	1.97 (1.45)	

^a^Expressed as mean (standard deviation). ^b^Expressed as median (range). N: number of patients.

**Table 2:** Estimates of population pharmacokinetic analysis of clozapine and norclozapine trough plasma levels in schizophrenic patients.

Parameter	Description	Final model estimate (RSE%)	Shrinkage	Bootstrap results (n=200)				
				Mean	RSE%	Median	95% CI	
*Population Mean*								
CLap_CZP_ (L/h)	Clozapine apparent elimination clearance in nonsmokers	28.1 (6)	-	27.8	5.6	27.8	24.7	31.1
CLap_CZP_ SMK (L/h)	Clozapine apparent elimination clearance in smokers	36.5 (8)	-	36.9	7.9	36.9	30.5	42.8
CLap_CZP_ (L/h)	Norclozapine apparent elimination clearance	53.6 (6)	-	53.5	6.5	53.5	46.7	60.2
F Luverina	Relative bioavailability of Luverina® versus Leponex®	0.892 (6)	-	0.895	6.8	0.896	0.769	1.02

*Between-subject CV*								
BSV CLap_CZP_ (%)		43.3 (12)	14	43.3	22	43.3	31.8	53.6
BSV CLap_NCZP_ (%)		49.9 (12)	18	50.3	26	50.4	35.9	64.2
BSV F (%)		43.6 (17)	26	43.9	34	43.9	24.7	58.9
cov CLap_CZP_ - CLap_NCZP_ (%)		55.7 (30)	-	34.1	26	34.1	24.0	43.2

*Residual variability*								
Proportional clozapine (%)		9.54 (21)	30	9.29	27	9.52	2.97	13.4
Proportional norclozapine (%)		15.3 (15)	22	15.3	16	15.2	10.8	20.3

## Data Availability

The data used to support the findings of this study are available from the corresponding author upon request.
